# ‘I Didn't Even Associate the Two Together at All’: A Qualitative Study of ‘Information Work’ Undertaken by Parents and Their Children With Epilepsy to Make Sense of Sleep and Seizures

**DOI:** 10.1111/hex.70763

**Published:** 2026-07-14

**Authors:** Holly Saron, Georgia Cook, Luci Wiggs, Kristina Dietz, Lucy Bray, Aiswarya Anilkumar, Tony Coffey, Paul Gringras, Wil Hardy, Dyfrig Hughes, Sylvine Lalnunhlimi, Christopher Morris, Deb K. Pal, Lucy Stibbs‐Eaton, Catherine Tudur Smith, Bernie Carter

**Affiliations:** ^1^ Faculty of Health, Social Care and Medicine Edge Hill University Ormskirk UK; ^2^ Oxford Institute of Applied Health Research, Faculty of Health Science and Technology Oxford Brookes University Oxford UK; ^3^ Centre for Psychological Research, Faculty of Health Science and Technology Oxford Brookes University Oxford UK; ^4^ MRC Centre for Neurodevelopmental Disorders & Basic and Clinical Neurosciences Department Institute of Psychiatry, Psychology & Neuroscience, King's College London London UK; ^5^ Liverpool Clinical Trials Centre, Institute of Population Health, Faculty of Health and Life Sciences University of Liverpool Liverpool UK; ^6^ Department of Children's Sleep Medicine Women's and Children's Institute, King's College London London UK; ^7^ Centre for Health Economics and Medicines Evaluation, North Wales Medical School, Ardudwy Bangor University Bangor Wales UK; ^8^ PenCRU (Peninsula Childhood Disability Research Unit) University of Exeter Medical School, University of Exeter Exeter UK

**Keywords:** child, epilepsy, information, parent, qualitative, sleep

## Abstract

**Background:**

Epilepsy in children presents challenges for families who must navigate the condition itself, along with complex medical information and the emotional realities of daily care. Underpinning a recent randomised control trial (CASTLE Sleep‐E) was the acknowledgement that sleep was a problem that had not been well addressed in other intervention studies in this population. The CASTLE Sleep‐E trial evaluated an online behavioural sleep intervention (CASTLE Online Sleep Intervention COSI]). An embedded qualitative study aimed to explore participating families' experiences of epilepsy, sleep and the trial itself. This paper focuses on the findings relating to participants' experiences of searching for information about sleep and epilepsy.

**Methods:**

Interpretive descriptive qualitative study embedded within a randomised controlled trial using remote interviews (October 2023 to February 2024) with parents (*n* = 22) and children (*n* = 22, aged 4–13 years) with epilepsy from both arms of the trial (COSI plus standard care or standard care alone). Data were reflexively thematically analysed. Patient and public involvement (parents and children) was central to both the main trial and this qualitative study.

**Findings:**

Five interrelated ‘information work’ themes were identified, four parent‐led themes: seeking information, surveillance as information work, sharing information and experiences, and struggling with information, and one child‐led theme: making sense of sleep and seizures.

**Conclusion:**

Many children with epilepsy and their parents become highly motivated information seekers; however, they face persistent gaps in credible, relevant and accessible guidance, particularly regarding the role of sleep in epilepsy. In response, they develop their own strategies for gathering, producing and interpreting information, often blending emotional labour, technology use and parental vigilance to improve sleep or address any sleep disturbance. These findings highlight a clear need for developing and implementing appropriate sleep support within paediatric epilepsy care.

**Patient or Public Contribution:**

Public and patient involvement (PPIE) was central to both the main trial and this qualitative study. A dedicated Advisory Panel (three children with epilepsy, ten parents of children with epilepsy and one adult who has lived with epilepsy since childhood) met regularly over a period of 6 years throughout the trial. Their insights and input shaped, informed and strengthened many aspects of our work from inception to dissemination, including refining the study aims and design, co‐developing materials and advising on data interpretation. Two of the Advisory Panel were co‐applicants on the programme grant. Advisory Panel members also contributed to the analysis and interpretation of qualitative data and reviewed the final manuscript for this paper, and their contributions are gratefully acknowledged. They were all compensated for their time and had any associated costs reimbursed. A more detailed account of PPIE is reported using the GRIPP2‐SF checklist.

## 1 Background

Childhood epilepsy is a complex neurodevelopmental disorder characterised by recurrent, unprovoked seizures, affecting approximately 1 in 200 children and young people aged 0–24 years in the United Kingdom [1], meaning around 112,000 children and young people are affected in the United Kingdom [2]. Childhood epilepsies manifest in different presentations and impacts on daily life ranging from complex syndromes through to milder forms. Globally, epilepsy is a leading cause of disability among all children and young people with a global prevalence estimate of 0.9% (95% UI: 0.8%–1.1%) [3]. Seizures, and side‐effects from their management, can impact children and young people in various ways, negatively affecting memory consolidation [4], school performance [5, 6] and sleep [5, 7, 8]. Childhood epilepsy, and its associated challenges, can also have a profound impact on families [9, 10]. For example, increased stress, anxiety, depression and sleep disturbances have all been identified in parents of children and young people with epilepsy [11]. For families and children, multifaceted challenges extend beyond medical management of epilepsy to a range of associated difficulties, including sleep disturbance; these deeply affect daily routines, schooling, social participation, emotional well‐being, quality of life and family functioning [12, 13, 14].

Of these various challenges, sleep problems (e.g., going to sleep, night‐waking) in children with epilepsy have been described as both a ‘top concern’ for children and parents [15] and identified as a core health outcome [16]. The complex bi‐directional relationship between sleep and epilepsy [13, 17, 18] has been described as a particularly vicious cycle [19], and the two have been described as ‘unfortunate bedfellows’ [17] as seizures can interrupt sleep and disrupted sleep can lower the threshold for seizures, exacerbating seizure frequency and severity. Importantly, both seizures and impaired sleep can independently impair cognitive functioning and negatively impact emotional regulation, thereby amplifying the overall burden of the condition [20, 21]. Nevertheless, research has tended to focus on seizure management with less attention paid to the management and impact of sleep on children and their families' lives and how families access appropriate information and support [13].

Parents, especially mothers, of children with chronic neurodevelopmental conditions are often the primary caregivers and become health information seekers, needing to access and understand complex medical information in order to manage treatments, understand risks and coordinate care [22]. However, the importance of access to accurate, relevant and understandable health information is not specific to parents of children with neurodevelopmental conditions as across the chronic illness literature evidence shows that information is critical not only for informed decision‐making [23] but also for fostering parental self‐efficacy, reducing uncertainty and self‐reported ‘fear’ [12] and mitigating the psychological distress associated with caring for a child with a chronic illness. Given that parents typically serve as the key information gatekeepers for their child [24], their ability to access, navigate and make sense of health information is central to the effective management of their child's condition and their overall health experiences. Less is known about how and where children with long‐term conditions themselves access information, although evidence exists about communication during clinical consultations [25, 26] and via online communities [27].

Obtaining relevant and trustworthy information has been identified as a significant challenge for many families affected by childhood epilepsy [28, 29, 30, 31, 32] even though there are some online informational materials (for parents and children) available from epilepsy support charities. Research with caregivers/parents highlights gaps such as lack of information [13] about comorbidities [30] and treatment options [32]. Additional barriers include inconsistent messaging, limited consultation time and healthcare professionals' variable expertise in discussing epilepsy‐related issues beyond seizure control, such as sleep problems [13]. This means parents must undertake ‘information work’ which was originally defined as ‘the quest for, the receiving of, and the passing of information’ [33, p. 10]. However, this definition can be elaborated to encompass the wider complex work of ‘attending to, making meaning of, communicating their bodily experiences, and attuning their daily lives around bodily experiences’ [34, p. 22].

Given the significance of sleep for children with epilepsy and their parents [15], the bi‐directional relationships between sleep and seizures [17] and the links between sleep and wider child and family functioning [12, 14, 35], it is important that families can access appropriate sleep information and support as needed. Limited existing evidence suggests that parents of children with epilepsy often struggle to access the information they want, in connection with various domains related to their child's epilepsy [13, 28, 29, 30, 31, 32]. However, there is currently a lack of contemporary robust qualitative research about how children with epilepsy and their parents seek information about epilepsy and sleep, what sources they access and the impact of the information they acquire.

The overall aim of the qualitative research, which formed part of the CASTLE Sleep‐E trial [18], was to explore participating families' experiences of epilepsy, sleep and the trial itself. This paper focuses on the data that explored how families seek, access, interpret and use information about epilepsy and sleep.

## 2 Methodology and Methods

### 2.1 Design

An embedded qualitative study, drawing on interpretive descriptive methodology [36], using remote semi‐structured interviews is reported in accordance with the Standards for Reporting Qualitative Research (SRQR) [37] and PPIE is reported in detail using the GRIPP2‐SF checklist (Supporting File S1) [38]. Ethics approval was obtained from the HRA East Midlands–Nottingham 1 REC (Ref: 20/EM/0205).

This qualitative study was conducted as part of the larger CASTLE Sleep‐E Trial [18], a multicentre, parallel‐group, unblinded randomised controlled trial in the United Kingdom (https://castlestudy.org.uk/) that aimed to evaluate the efficacy of an online sleep intervention (COSI) tailored for children with epilepsy (Supporting File S2: Overview of Trial). The trial was registered prospectively on the International Standard Randomised Controlled Trial Number Registry (ISRCTN13202325), the study protocol [39] and primary findings have been published elsewhere [18]. The trial opened to recruitment on 11/05/2022 and recruitment closed on 18/10/2023.

### 2.2 Setting and Participants

Primary caregivers (referred to as parents) and children were recruited to the main trial through 26 NHS paediatric epilepsy outpatient clinics. Eligible children were aged 4–12 years at the time of recruitment, lived in the United Kingdom, had a clinician‐confirmed diagnosis of childhood epilepsy and a parent‐reported child‐sleep problem. Families were excluded if the child had a moderate to severe learning disability or if the parent lacked sufficient English to understand study materials. These criteria were created to ensure participants would be able to effectively utilise the COSI resources in the trial as these were only available in English. This inevitably restricted the diversity of families who were eligible to participate in the embedded qualitative study.

### 2.3 Ethics

Tailored information sheets were provided for both parents and children. Informed consent was collected for all participants via the person with parental responsibility (or as appropriate in the devolved nations). As the inclusion criteria were children aged ≥ 4 to ≤ 13, assent was also sought for children aged ≥ 7 (if they wished to provide it). Consent was written via an e‐consent format following discussion about the trial and qualitative component with the local research team at the hospital; all people (e.g., research nurses) taking consent were trained in taking consent/assent and had received training specific to the trial. Declining participation in the qualitative interviews did not affect eligibility for the main trial.

### 2.4 Interviews With Parents and Children

Parents and/or children who expressed interest in taking part in the qualitative component were invited to participate in remote semi‐structured audio or audio‐video interviews undertaken by H.S. (female, white, social sciences academic with experience interviewing children and families). The interviews were conducted by phone or via Microsoft Teams at two timepoints (TPs) following trial data collection: TP1 (3–4 months) and TP2 (6–7 months) after randomisation with the aim being to see how, if at all, perspectives shifted during the study. Interview topic guides including activity booklets for children were sent to families about a week before the scheduled interview to give them time to prepare. Parents could make notes on their interview guides, and children could add drawings or complete the tasks in the activity booklets and then use these as prompts in the interviews. In the audio‐video interviews, some of the children showed the interviewer their drawings (Supporting File S3: Examples of guides and booklets). Interviews were conducted between October 2023 and February 2024.

The interviews with parents opened with the request ‘Please tell me about you, your family's and your child's experiences of living with epilepsy’ (prompts included changes to their lives, management of their child's seizures, feelings about epilepsy and challenges faced) (Supporting File S4: Interview guide). We then moved on to asking them to tell us about any issues they had with their own and their child's sleep (prompts included bedtimes, importance of sleep, sleep disruptions, impacts and strategies, accessing information and decision‐making). For parents using COSI, we asked about their own and their child's experience of using COSI (prompts included motivation, ease of use and how COSI could be improved). The interviews with children were supported by the information they had shared in the activity booklets. The interviews explored how they felt about their epilepsy, and what things were changed by having epilepsy. Sleep‐related questions included ease/difficulty going to sleep, staying asleep, waking up in the morning, what helped them get to sleep and how they had found monitoring their sleep as part of the trial.

Children could be interviewed separately or jointly with their parents, depending on family preference and the child's age and comfort. In all but one case, dyadic interviews were undertaken with parents and children aged 4–13 years; one parent–child dyad was interviewed separately. Although our stance was that children are competent, agentic and capable of participating in research and sharing their experiences and ideas, we were sensitive to the power asymmetries and relational and interdependent influences [40] that can exist in research with children (e.g., researcher–child and parent–child). Parental presence may act in different ways (e.g., suppress, coerce and support) a child's ability to express their views [41]. A child's parent, even with good intention, may shut down, override or present their allegedly more authentic adult perspective. Children may be unwilling to share feelings or ideas that they see as transgressive, sensitive or which may upset the parent; alternatively, parents can support the creation of an environment in which a child feels comfortable disclosing thoughts, feelings and experiences [42]. Being sensitive to how these relational, contextual and other interdependent influences play out is perhaps trickier or different when using remote interviews (e.g., researcher has little control over the environment and may miss some nuances and/or interference by the parent that limit a child's response) [40, 41, 42]. By being alert to such influences and experienced in undertaking remote interviews with children and parents, we aimed to ensure that the child's views were central within the interviews.

### 2.5 Data Analysis

All interviews were audio‐recorded and transcribed verbatim by an approved (University and Sponsor) transcription service and then anonymised and checked by members of the analysis team. While member checking is sometimes used to enhance validity [43], the authors chose not to pursue this step to avoid overburdening families in the final weeks of trial participation; some parents had said that trial participation was quite intensive.

Data were reflexively thematically analysed following Braun and Clarke's [44, 45] six‐phase approach. We adopted an experiential orientation to the data as we were interested in the lived experiences and knowledge of our participants. All analyses were conducted manually without qualitative software. The core qualitative analysis team (B.C., H.S., G.C., L.W. and K.D.) read transcripts multiple times (‘familiarisation’) before inductively generating initial impressions of the data and suggestions for codes. Then H.S. and B.C. worked more intensely with the transcripts, ‘generating initial codes’ (e.g., ‘questions’, ‘challenge’, ‘search’ and ‘source’), creating mind‐maps, writing notes and ‘searching for’ and collating these into potential themes (e.g., ‘struggle’, ‘vigilance’) that reflected parents' and children's sleep and information‐related experiences. These initial themes were shared with the Advisory Panel to check resonance and interpretive accuracy. We deemed this engagement with the Advisory Panel to be crucial as it helped ensure that our meaning‐making (interpretation) had coherence with how parents with lived experience made sense of our themes and ideas. This is a step that is not always woven into data analysis, but one we believe adds a level of robustness and grounding. Further iterative dives back into the transcripts allowed ‘reviewing’ (e.g., shift from ‘vigilance’ to ‘surveillance’) and then ‘defining and naming’ of the near‐final themes; gradually building a deeper interpretive appreciation of the data and supporting the central concept of ‘information work’. The themes were shared and considered by the rest of the core analysis team, refined, ‘defined and named’. In the final phase (‘writing up’), representative quotations were selected to illustrate key points. Trustworthiness (credibility, transferability, dependability and confirmability) [46] was addressed.

The analysis team were ethnically white British, academics with children's nursing, psychology or human geography backgrounds. The diversity in disciplinary background resulted in interesting debates about language, interpretation and meaning; these were documented as reflectixe notes. These debates, along with the challenges and advice from the Advisory Panel, gave us confidence that we were being reflexive in our engagement with the data. We aimed for ‘reflexive openness’ [47] and we acknowledged our subjectivities and the context of our study and were self‐conscious and collaborative in how we critiqued, appraised and evaluated our influences across the duration of the research [48].

Quotations are referenced using a participant number and code (e.g., M^1^/F^2^/B^3^/G^4^) indicating role, Mother (M), Father (F), Boy (B) or Girl (G) and trial allocation (COSI [standard care plus sleep intervention]) or standard care (SC), as well as engagement status for those assigned to COSI (^E^ = Engager; ^NE^ = Non‐engager). Engagement was determined by whether they accessed one or more of the modules that included advice about child‐sleep behaviour change strategies, on one or more occasions. TP2 (Timepoint 2) indicates that the interview was undertaken 6 months after randomisation; all other interviews were TP1 (3 months after randomisation); to reduce length of label we do not include TP1 as part of the label. So, M^16^, COSI^E^, denotes a mother in the COSI arm who engaged and whose data reflects TP1. Only those families randomised early enough in the trial were eligible for TP2 within the trial window.

## 3 Findings

### 3.1 Participants

Forty‐three of the 85 families who participated in the trial agreed to be contacted by the qualitative team; 16 of these families did not respond, and five declined to be interviewed. Those who declined stated personal or family factors and parental preference (e.g., ‘personal circumstances’, ‘unavailable due to holiday’) or child preference (‘not keen’). Thus, of those who had expressed initial interest, 22 families (19 mothers, 3 fathers, 12 boys and 10 girls, age 4–13 years) participated in interviews at TP1; one child recruited to the trial aged 12 years was 13 at the time of interview (see Table 1 for details of TP1 and TP2 participants).

**Table 1:** Overview of demographic information.

Timepoint 1 (*n* = 22)		Timepoint 2 (*n* = 3)	
*Standard care (SC) (n = 13)*		*Standard care (SC) (n = 2)*	
Mother	*n* = 12	Mother	*n* = 2
Father	*n* = 1	Father	*n* = 0
Children (4–13 years, mean 9.3 years)		Children (12 & 13 years, mean 12.5 years)	
• Boy	*n* = 7	• Boy	*n* = 0
• Girl	*n* = 6	• Girl	*n* = 2
*COSI engagers (COSI^E^) (n = 6)*		*COSI engager (COSI^E^) (n = 1)*	
Mother	*n* = 4	Mother	*n* = 1
Father	*n* = 2	Father	*n* = 0
Children (8–12 years, mean 9.8 years)		Children (12 years)	
• Boy	*n* = 4	• Boy	*n* = 1
• Girl	*n* = 2	• Girl	*n* = 0
*COSI non‐engagers (COSI^NE^) (n = 3)*			
Mother	*n* = 3		
Father	*n* = 0		
Children (10–13 years, mean 11.3 years)			
• Boy	*n* = 1		
• Girl	*n* = 2		

All families identified as White. Families participated from six regions in the United Kingdom: most from the East of England (*n* = 6) and Yorkshire and the Humber (*n* = 5) and then Wales (*n* = 2), East Midlands (*n* = 2), North West (*n* = 2), South East (*n* = 2), London (*n* = 2) and the North East (*n* = 1).

Area deprivation derived from residential postcode was grouped into quintiles (Q); participants were represented across all categories except Q2. The highest proportions were found in both the most deprived quintile (Q1; 36.4%) and the mid‐range quintile (Q3; 36.4%), with a further 22.7% falling into the least deprived quintile (Q5) (Table 2).

**Table 2:** Summary of deprivation by area of residence (indices of multiple deprivation quintiles (*N* = 22).

Quintile	*n* (%)
1 (most deprived)	8 (36.4%)
2	0
3	8 (36.4%)
4	1 (4.5%)
5 (least deprived)	5 (22.7%)

The timing of the child's first parent‐reported seizure in relation to their randomisation in the study ranged from 8 years to 6 months, and all but two parents had at least 12 months experience in managing their child's seizures and sleep issues. The most common parent‐reported epilepsy diagnoses were genetic generalised epilepsies (*n* = 7); focal epilepsy not otherwise subclassified (*n* = 5); self‐limited epilepsy with centrotemporal spikes (SeLECTs)/benign epilepsy with centrotemporal spikes (BECTS)/Rolandic epilepsy (*n* = 5), and other epilepsies (*n* = 5) (focal epilepsy/Lennox–Gastaut syndrome, nocturnal epilepsy, likely genetic epilepsy syndrome, SCN1A mutation with prolonged atypical febrile and afebrile convulsions, and not specified).

Although our primary focus is reporting qualitative data, it is valuable to provide context in relation to the score that indicates parent‐reported sleeplessness problems (settling, night‐waking, early‐morning waking and co‐sleeping); this was assessed via a Composite Sleep Disturbance Index based on questionnaire responses, with possible scores ranging from 0 to 14, with higher scores indicating increased problems. A score of 7 or more would indicate the presence of at least mild sleeplessness [49]. For children reported in this paper, the mean score (and standard deviation [sd]) was 5.9 (sd 3.6). Only six children scored at or above the threshold for mild sleeplessness (indicating that 16 children did not have significant sleeplessness problems) (Supporting File S5: Overview of sleep measure).

Only three families continued to TP2 due to the end date of the trial. Of the 22 families, 13 were in the SC arm and 9 in the COSI arm (6 engagers, 3 non‐engagers). Two of the three COSI^NE^ cited family crises as reasons for non‐engagement with the resource.

### 3.2 Thematic Findings

**Figure 1:**
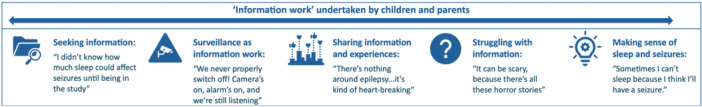
Information work undertaken by children and parents.

One overarching theme (‘information work’) and five interconnected sub‐themes were generated (Figure 1), four reflecting how parents sought information, turned to surveillance as a way of knowing, wanted to share and exchange insights and struggled with the uncertainty surrounding their child's epilepsy. The fifth theme focuses on children's own sense‐making about sleep and seizures.

#### 3.2.1 Seeking Information: ‘I Didn't Know How Much Sleep Could Affect Seizures Until Being in the Study’

Before their child's diagnosis, most parents had little understanding of epilepsy.

After diagnosis, parents described a strong motivation to seek information but found limited or inconsistent guidance, and this could be frustrating. Some parents felt supported by professionals—typically epilepsy nurses—to seek and gain information. However, parents often turned to the internet for information or combined online material with medical input, for example, ‘read[ing] up on Google, and off her epilepsy consultant’ (M^13^,SC,TP2). Sleep‐related information was often sought from the internet as it was not something routinely discussed in the clinic. One mother noted she was

aware that lack of sleep can be a trigger [for seizures]. I've read loads of stuff online because I just have to know everything.

(M^17^,COSI^NE^)

Epilepsy‐specific online parents' forums were sources of peer‐provided information. Some parents were positive about these sources as they were able to gain ‘amazing help’ (M^12^,SC) and relevant information. However, experiences with online information varied widely and did not always provide the answers parents were searching for such as practical advice about installing ‘blackout blind[s]’ (M^12^,SC) or specific sleep‐related information relating to ‘helping him to get up in the morning… rather than getting him to sleep, the other end of it’ (M^16^,COSI^E^).

Typically, parents talked of how their understanding of how sleep affected epilepsy came through lived experience rather than professional guidance. One father reflected that his daughter's seizures seemed more frequent or intense when she was tired and having ‘less than four hours a night and very broken sleep’ (F^8^,COSI^E^), making the link between sleep and how her epilepsy manifested.

For some families, the decision to join the CASTLE Sleep‐E trial was driven by a desire to learn more about their child's condition, and participation in the study provided the first structured explanation of sleep's role in epilepsy. Many had not previously known of the association between sleep and seizures; one mother explained:

we didn't get any information around sleep and epilepsy at all… The trial was the first time we'd really zoned in on [sleep and epilepsy].

(M^17^,COSI^N^)

Overall, the parents' accounts revealed them to be proactive information seekers. However, from their perspective, sleep‐specific knowledge was rarely reported as being available or gained through routine care.

#### 3.2.2 Surveillance as Information Work: ‘We Never Properly Switch Off! The Camera's On, the Alarm's On, and We're Still Listening’

When parents had not received clear or consistent professional advice, they developed their own strategies to feel informed and in control. Parents described the lengths they would go to understand their child's epilepsy, seizures and sleep patterns. Their actions often aimed at mitigating risks such as ‘sleeping on [child's] bedroom floor for months’ (M^7^,COSI^NE^) or purchasing equipment to alert them to night‐time seizures including ‘alarms’ (M^15^,SC), ‘cameras’ (M^12^,SC), ‘sleep monitors and sensor mats’ (M^7^,COSI^NE^,TP1) and ‘night watchers’ (M^3^,SC). These practices were as much as both knowing as protecting, reflecting efforts to gather individualised real‐time information that was good evidence that they could share with professionals.

Technology became a tangible form of reassurance, symbolising both agency and anxiety. Some parents (and at least one child) used sleep tracking apps in their own fitness trackers or smart watches. The importance of giving their child an active role in managing risk was evident, for example, one mother described how she had bought an alarm:

so that she [daughter] could set it off if she felt she was having a seizure and we weren't in the room.

(M^15^,SC)

Parents described the constant tension between the need for reassurance and the exhaustion of constant vigilance, explaining how nightly routines of watching and listening for seizure activity left them drained and unable to rest. Even when things were going well, vigilance was maintained, explaining they never ‘properly switch off’ (M^12^,SC); this took a toll on their own sleep:

ever since the seizures, I'm such a light sleeper now…one eye open. Even if I hear something and it's not her, I'm still getting up and checking… I'm probably only getting three or four hours [sleep] at most.

(F^8^,COSI^E^)

Despite profound fatigue, surveillance—a form of hypervigilance and risk management—was described as providing parents with a sense of reassurance that they were doing the best for their child.

#### 3.2.3 Sharing Information and Experiences: ‘There's Nothing Around Epilepsy… and It's Kind of Heart‐Breaking’

Parents were emphatic about the value of peer support through shared experiences and mutual understanding, both for themselves and for their children. Despite some accessing online support groups, they consistently described what they perceived as the absence of structured opportunities to connect with others facing similar sleep‐related challenges recalling that ‘there's just nowhere to turn to… and it's kind of heart‐breaking’ (M^7^,COSI^NE^).

This lack of community, coupled with limited information from professionals particularly in relation to sleep, left parents feeling isolated in their experiences and unsure how their child's situation compared with others. As one mother explained,

you don't really talk to other parents [of children with epilepsy] about it… no one else really gets what it's like at night.

(M^12^,SC)

Several parents saw the trial as ‘good thing’ and an opportunity to become part of an altruistic community in which they could share information and experiences and help other parents.

Sharing and wanting to share was an unmet need, with families compensating for the absence of formal support networks by implementing things that make sense to them.

#### 3.2.4 Struggling With Information: ‘It Doesn't Quite Feel Like My Experience’

Some advice provided by professionals was conflicting, ‘one person says one thing and another person says another thing’ (M^11^,COSI^E^). In the context of limited and/or conflicting advice about sleep, participation in the trial (regardless of the arm they were randomised to) was perceived by most participants as an opportunity to access useful and authoritative information, support and answers. One mother recalled:

we'd had such hard times with sleep… our epilepsy nurse immediately asked if we wanted to join the study.

(M^18^,COSI^E^)

The perceived absence of information provided by health professionals or those with lived experience triggered some parents to turn to less controlled information sources such as social media. Although some parents benefited from the information available from using social media, these sources rather than providing reassurance could be perceived as ‘voyeuristic’ (M^19^,SC) or frightening:

because there's all these horror stories… maybe awareness should be more moderated.

(M^2^,SC)

Information and experiences that were shared via social media (e.g., Facebook) by parents in apparently worse situations than their own caused parents to disengage, ‘I looked once and never again. I don't want to stress about it’ (M^20^,SC), because they perceived their child was not ‘suffering like that [child]’ (M^17^,COSI^NE^). Additionally, advice received from social media did not necessarily have positive outcomes; one mother talked of experimenting with online or over‐the‐counter sleep aids that did not work, including ‘sleep gummy bears off Amazon’ and ‘kids’ sleep patches’ (M^6^,SC).

The emotional and practical consequences of inconsistent, overwhelming or distressing advice, particularly from unregulated sources, often added to parents' confusion and anxiety.

#### 3.2.5 Making Sense of Sleep and Seizures: ‘Sometimes I Can't Sleep Because I Think I'll Have a Seizure’

Children's accounts offered a distinct but complementary perspective on sleep, revealing how their awareness and sense‐making was shaped by both their lived experience and parental influence. Their involvement in information seeking was less direct than their parents, generally relying on parents to interpret and manage information on their behalf. Typically, their understanding of their own sleep issues and those of their wider family evolved as time went on.

For younger children, sleep was inseparable from emotional security. Many expressed a need for proximity and reassurance, finding comfort in the presence of a parent, explaining ‘I hold Daddy's hand…having Mummy and Daddy there helps me fall asleep’ (B^4^,6^yrs^,SC).

For the older children who had more lived experience of having epilepsy, their reflections became more analytical, suggesting a deepening awareness and understanding of the importance of sleep. Some children recognised the link between sleep deprivation and seizures, noting that ‘when I'm tired, I have more seizures. When I'm not tired, I'm fine’ (G^8^,11^yrs^,COSI^E^). Others described patterns of tiredness and energy explaining:

sometimes at night I have too much energy, if I haven't gotten rid of that energy, it's hard to sleep, then I can get really tired in the morning.

(B^3^,12^yrs^,COSI^NE^)

Occasionally children searched online for information demonstrating curiosity and a desire for answers, ‘sometimes my brain just gets curious and I type it up into Google… on my phone’ (G^21^,11^yrs^,COSI^NE^).

Typically, children were anxious about sleep. Several children spoke fearfully about night‐time seizures, worrying about prospective seizures that might not happen since ‘I don't know if I'm going to have a seizure that night’ (B^12^,11^yrs^,SC). Such fears often shaped bedtime routines and emotional states. Some children used personalised strategies for relaxation or distraction such as playing ‘my wave music before bed’ (G^13^,12^yrs^,SC) or sensory aids such as ‘blankets’ (G^10^,12^yrs^,SC).

Older children expressed a growing desire for autonomy and control over their bedtime routines (e.g., being able to decide when to go to bed). Constant parental night‐time monitoring created tensions around privacy; one girl expressed resentment about the camera in her room because it meant ‘there is someone watching me’ (G^10^,12^yrs^,SC).

Their accounts demonstrate a developmental shift from dependency and reliance on their parents' vigilance toward aspiration for privacy, personal responsibility and self‐management. However, this was reliant on them being informed and developing confidence in living with epilepsy.

## 4 Discussion

Our paper describes how mothers, fathers and children engage in everyday ‘information work’ [34], by seeking, sharing, struggling, interpreting and producing knowledge through practices such as surveillance, to make sense of childhood epilepsy, seizures and their relationship with sleep. Our work is innovative in that the concept of ‘information work’ has not previously been used in this population. Further, we have shown that the ‘information work’ undertaken by parents extended beyond just questing for, receiving and passing on information [33] to more sophisticated aspects of ‘information work’ [34] in terms of connection [50], meaning‐making and attuning their (night‐time) lives around their concerns and strategies relating to sleep and seizures. As seen elsewhere, this form of information work was not optional for parents [51]; it was a complex, necessary additional and embodied dimension of parenting that they undertook to keep their child safe. Core to information work are affordances (social, physical and material opportunities for gaining information) [52] that support decision‐making. The importance for parents of meaning‐making in relation to their child's epilepsy and sleep, exposes the importance of the health professionals to create the opportunities and affordances necessary to meet their information needs when they engage with parents. However, the focus within clinic settings is often on medication and seizure activity, both essential elements for review, but not necessarily creating opportunities for a wider consideration of the child's health and well‐being. Although both NICE guidelines [53, 54] and the RCPCH [55] acknowledge the importance of multi‐disciplinary integrated services that provide holistic support including ‘vigilance on mental health’, there is little mention of promoting sleep hygiene or supportive sleep interventions. However, the ‘care bundle’ for children and young people with epilepsy [56] does recommend that comprehensive care plans should address sleep. Epilepsy nurse specialists are perhaps the best positioned members of the multidisciplinary team to engage with parents about sleep‐related concerns, creating a climate of opportunity for learning and understanding. However, inequities in service provision mean not all children and parents have access to epilepsy specialist nurses [57].

Prior to their participation in the CASTLE Sleep‐E trial, some parents had not known about the bi‐directional relationship between sleep and epilepsy; they ‘didn't even associate the two together at all’. This gap in what could be considered core knowledge about sleep and epilepsy could result from information being inconsistently available, emotionally charged and often acquired through lived experience and searching online rather than clinical guidance provided by professionals. This aligns with the reported dearth of reliable, accessible family‐oriented information about management of childhood epilepsy and specifically sleep in childhood epilepsy, as reported in other studies [13, 58]. As seen in other studies, sleep information was sought in different settings, in clinics from health professionals as well as from less controlled and more unreliable settings such as online forums [27]. Whilst online communities can offer emotional support and foster a sense of connection, there are attendant risks such as erroneous or misleading health advice. Although we did not ask parents if they communicated with their health professionals about questions or worries arising from engagement with online communities, other studies suggest that parents do not share web‐based information with professionals [59]. This creates potential for parents to translate and adopt poorly evidenced and fragmented information into their child's night‐time routines; at best, these may not be harmful, at worse advice may be unsafe. Although not widely available/used at the time of our data collection, generative AI holds positive promise in terms of responding to parents' and/or children's search for information. However, errors such as AI hallucinations can present misleading information with an apparent high level of confidence [60]; this is likely to be an increasing future challenge that needs to be addressed in practice and research related to health‐related information seeking.

Parents' surveillance of and vigilance over their child's bedtime and sleep is part of their attunement to their child, part of their night‐time routine of trying to keep their child safe [61, 62]. Their vigilance reflected their fears and knowledge about night‐time seizures and the nature of the information they had acquired. Frightening online stories shared by other parents can exacerbate fears; we saw this in parents who reported distress from exposure to ‘worse than my child’ stories. Increasing anxiety resulting from distressing online information [59] is likely to heighten vigilance and the ‘emotion work’ (managing feelings and emotions) [51], an often over‐looked aspect of the ‘information work’, that parents are already undertaking. This needs to be acknowledged and addressed in practice. However, this vigilance sometimes created tension between parents and their child whose desire for privacy and autonomy increased as they got older. Health professionals could ease some of the challenges parents and children face in relation to ‘information work’ by providing tailored information [63] to improve sleep, reduce the risk of seizures and ensure that the level of vigilance is appropriate and acceptable.

Despite the evidence of epilepsy disrupting both children's and parents' sleep [11, 64], and a report from a policy lab on improving sleep of children with epilepsy noting that ‘sleep is everybody's business’ [15, p. 4], our parents, as seen in other studies, reported struggling to access relevant information from health professionals about epilepsy [12, 29, 65, 66] and its impact on sleep [13]. Although some participants spoke positively about the support received from epilepsy nurses, others felt their information needs in terms of informational and emotional support had not been sufficiently well attended to; other studies reveal similar issues in terms of a shortfall in effective communication and information sharing [31, 67]. ‘Information work’ for parents is complex and individual to each family and closely linked to their everyday realities [34]; the challenge for health professionals lies in delivering the right information about sleep at the right time. Our parents indicated that the ‘right time’ was after they had absorbed initial information about diagnosis, seizure management and treatment, aligning with other work suggesting that parents' concerns about sleep assume higher priority about 12 months post diagnosis as part of concerns about their ‘child's global functioning and well‐being outcomes’ [30, p. 6]. For trusted professionals to be able to deliver the ‘right information’, they require appropriate evidence‐based resources and training [15]; this has the potential to enhance parents' self‐efficacy in managing their child's epilepsy [68] and sleep. However, in the current climate where services are stretched delivering core care, opportunities for staff to enhance clinical communication skills or develop their own expertise in supporting positive sleep practices may be limited.

In the absence of parents having access to timely and appropriate professional information, they seek other trustworthy sources; our parents saw the trial as an opportunity to engage with professional experts who could address some of their epilepsy‐related knowledge gaps. For some parents, the CASTLE Sleep‐E trial [18], regardless of the arm or level of engagement, emerged as a valuable—sometimes the first structured and reliable—source of health‐related knowledge of the link between sleep and seizures. Typically, clinical trials have a relatively narrow focus on specific aims and outcomes. However, our findings suggest that trials can also serve a broader, more impactful role prompting reflection and learning for participants. However, relying on such opportunities is insufficient to ensure equitable knowledge and skill development. Rather than the serendipity of participating in a trial, children and parents need access to credible, effective and accessible educational resources; the evidence is that access to appropriate information and support does increase knowledge, empowerment and potentially (self)‐management [69]. If we are to reduce the challenges associated with the ‘information work’ undertaken by parents and children and improve their sleep, then providing information about the association between sleep and seizures and how to manage sleep really is ‘everybody's business’ [15, p. 4]. Parental ‘information work’ is likely to be eased by health professionals who are confident, capable and responsible for creating affordance‐rich settings in which parents are comfortable seeking and discussing sleep‐related information relevant to their situation. One such affordance could be the use of discussion sheet/framework [54] the parents and child complete prior to an appointment and share with professionals.

### 4.1 Limitations

Although around 26% of trial families participated in the qualitative study, the findings should be considered in relation to the following limitations in terms of transferability. Despite inclusion of a range of families within the trial (*n* = 85) across multiple NHS sites in the United Kingdom, participation in the trial was necessarily limited to those with sufficient English proficiency to engage with intervention materials and complete interviews and those with sufficient motivation and resources (e.g., time) to participate. All parents we interviewed identified as White, broadly reflecting the trial sample where 89.41% trial participants identified as White. Our study sample under‐represents those from ethnic minority backgrounds and those not fluent in English; such a lack of diversity has been widely reported as an issue across healthcare literature [70, 71, 72] and within research participation guidelines [73] as under‐representation results in health inequalities and services/interventions not meeting their needs. In our study, this lack of representation is likely to mean that our findings do not represent those whose information work could be even more challenging due to cultural, socio‐economic, linguistic and other influences. Future work should critically consider the influence of ethnic and cultural diversity. Additionally, fewer fathers than mothers participated; this is not unusual in family‐based studies where fathers tend to have lower participation rates as mothers often take on primary caregiving and health communication roles. Additionally, only two children had had their first seizure within 12 months of trial participation and based on parent report, 16 children did not have significant sleeplessness problems according to the Composite Sleep Disturbance Index, so our families were experienced in managing, perhaps even habituated to sleep disturbance. As a result, the findings on information seeking and sleep management may overrepresent the views of White mothers of children who have had seizures for some years and who are from families with sufficient motivation and resources to participate in a trial. A further limitation is that only three families participated in TP2 interviews, primarily due to the delays in trial recruitment resulting in only a smaller subset of families being eligible to have a TP2 assessment (6 months post‐intervention) before the close of the trial. Inevitably, this prevented us from undertaking comparison between TPs or presenting any longitudinal findings.

## 5 Conclusion

Children with epilepsy and their parents engage in, and struggle with, complex ‘information work’ to support sleep. Findings highlight key areas of information provision that parents perceive to be missing and how parents and children try to fill information gaps. Results emphasise the need for accessible, trustworthy, child and family‐centred information from healthcare professionals that addresses the fears, concerns and practical sleep and epilepsy‐related problems children and their parents face. At a minimum, parents need to be aware of the bi‐directional relationship between sleep and seizures. However, precise information needs will vary from family to family, as will preferences for where they get their information. For example, some parents find reassurance in sharing and exchanging experiences with others, and tolerance of ‘better/worse than my child’ stories; others find this overwhelming and/or distressing. Information sharing about child sleep therefore needs to be adaptable, sensitive to individual differences and coping styles and to address the pragmatic issues, and psychosocial and emotional dimensions, as well as medical components related to epilepsy management. Future interventions must recognise children and parents not as passive recipients of information but as co‐creators and active knowledge‐makers whose experience and expertise are central to effective epilepsy care and for improving the lives and experiences of children with epilepsy and their parents.

## Author Contributions

**Holly Saron:** conceptualization, methodology, investigation, writing – original draft, writing – review and editing, formal analysis. **Georgia Cook:** conceptualization, writing – review and editing, methodology. **Luci Wiggs:** conceptualization, methodology, writing – review and editing. **Kristina Dietz:** writing – review and editing, project administration, formal analysis. **Lucy Bray:** conceptualization, funding acquisition, writing – review and editing, methodology. **Aiswarya Anilkumar:** project administration, writing – review and editing. **Tony Coffey:** project administration, writing – review and editing. **Paul Gringras:** conceptualization, funding acquisition, writing – review and editing. **Wil Hardy:** writing – review and editing. **Dyfrig Hughes:** writing – review and editing. **Sylvine Lalnunhlimi:** project administration, writing – review and editing. **Christopher Morris:** conceptualization, writing – review and editing, funding acquisition. **Deb K. Pal:** conceptualization, writing – review and editing, data curation. **Lucy Stibbs‐Eaton:** writing – review and editing. **Catherine Tudur Smith:** writing – review and editing. **Bernie Carter:** conceptualization, investigation, methodology, funding acquisition, writing – original draft, writing – review and editing, validation, visualization, formal analysis, supervision.

## Ethics Statement

Ethics approval was obtained from the HRA East Midlands–Nottingham 1 REC (Ref: 20/EM/0205). We obtained remote informed consent (assent, as appropriate) from all participants. Study participation was voluntary.

## Conflicts of Interest

The authors declare no conflicts of interest.

## Supporting information


Supporting File 1

Supporting File 2

Supporting File 3

Supporting File 4

Supporting File 5

## Data Availability

The data that support the findings of this study are available on request from the corresponding author. The data are not publicly available due to privacy or ethical restrictions.
