# Supplementary material for: ‘I Didn't Even Associate the Two Together at All’: A Qualitative Study of ‘Information Work’ Undertaken by Parents and Their Children With Epilepsy to Make Sense of Sleep and Seizures
Source: Health Expect. 2026 Jul 14;29(4):e70763. doi: 10.1111/hex.70763 (PMC13366387; doi:10.1111/hex.70763)
Supplement: Supplementary file 1 — Supporting File 1 [file HEX-29-e70763-s004.pdf]

Supporting file 3: Overview of PPIE described using GRIPP2-SF (reporting checklist to improve reporting of patient and public involvement in research)*

Topic	Item
1. Aim	Public and patient involvement (PPIE) was key to the study and this took the form of our Advisory Panel (AP).<br><br>The AP was supported to bring their own lived experience and expertise to the CASTLE Sleep-E trial and the qualitative study embedded within it. The aims of the close engagement with the AP were to:<br>• shape the study aims and design,<br>• co-develop materials,<br>• advise on data interpretation,<br>• advise on dissemination.
2. Methods	The AP (three children with epilepsy, ten parents of children with epilepsy, and one adult who had lived with epilepsy since childhood) met regularly with a member of the research team (LB), mostly online with occasional in-person meetings over a period of six years. The group also maintained an active online chat for ongoing communication. They helped design study materials including online videos, information sheets and infographics to help families who would be recruited understand the study. They advised on recruitment approaches including inclusion and exclusion criteria and the questions addressed within the interview topic guide. They also shared their views on developing thematic findings and reviewed and provided feedback on this paper. Steps were taken to ensure that the group had access to the necessary IT and any additional resources they required, for example posting materials when email was not suitable. All members were compensated for their time and reimbursed for any associated costs.
3. Study Results	The PPI strengthened the study in many ways. Their lived experience helped shape the focus on sleep as an often-forgotten part of living with epilepsy. They ensured that recruitment approaches and data collection procedures were considerate of family's busy lives and information was accessible, engaging and helped families make an informed decision about taking part. They provided an ongoing critical perspective on the value of the qualitative study and the implications of the findings to families. Their input into data interpretation led to additional exploration of the data.<br>Young people with epilepsy were not involved in the interpretation of the qualitative data due to commitments at the time of analysis.
4. Discussions and Conclusions	Public and patient involvement (PPI) has had and will continue to have a meaningful influence on the direction, delivery and dissemination of the study, based on the impacts in the study results section.<br>As the AP worked together for six years this enabled trusting and positive relationships to be fostered over time between members and with the facilitator. This was important as AP members became more confident over time to challenge researchers, question decisions and contribute to the qualitative elements of the study.<br>Members of the AP reviewed the manuscript of the Information and Sleep paper providing additional insights into the themes and discussion.

	One AP member stated that the paper was “a good read...quite emotional in parts”.
5. Reflections/critical perspective	Involving the advisory panel from the very beginning of the study and including two members as co-applicants on the programme grant, helped to establish a genuine partnership approach. Panel members contributed to key decisions, influenced the focus and direction of the project and embedded their lived experience at the heart of the study. The ongoing relationships formed within the AP, supported by the active online chat, helped to build trust and rapport among members, which in turn sustained engagement, and ensured that lived experience continued to inform the study throughout its duration.<br>The AP members involved were heavily invested in the study whilst often still facing challenges within their own families in the management of epilepsy. This meant that care and regular check-ins were important in ensuring that AP members were supported.

* Staniszewska S, Brett J, Simera I, et al. (2017) GRIPP2 reporting checklists: tools to improve reporting of patient and public involvement in research. *BMJ* 358: j3453
