# Supplementary material for: ‘I Didn't Even Associate the Two Together at All’: A Qualitative Study of ‘Information Work’ Undertaken by Parents and Their Children With Epilepsy to Make Sense of Sleep and Seizures
Source: Health Expect. 2026 Jul 14;29(4):e70763. doi: 10.1111/hex.70763 (PMC13366387; doi:10.1111/hex.70763)
Supplement: Supplementary file 2 — Supporting File 2 [file HEX-29-e70763-s005.pdf]

Supporting file 2: Overview of the CASTLE Sleep-E trial

**Design**	• CASTLE Sleep-E was a multicentre, parallel-group, unblinded, randomised controlled trial that evaluated the clinical and cost effectiveness of COSI (an online sleep intervention for parents of children with epilepsy).<br>• The Children's Sleep Habits Questionnaire (CSHQ) (at 3 months) was the primary outcome. Cost-effectiveness was estimated at six months. Intention to treat analyses were undertaken.
**Recruitment**	• Eighty-five children (aged 4-12 years) with epilepsy and sleep problems were recruited via 26 UK outpatient clinics.<br>• They were randomly (1:1) assigned via a computer-generated minimisation algorithm.<br>• The 85 children were assigned to standard care (SC) (n=42) and SC+COSI (n=43).
**Results**	• The adjusted mean CSHQ difference between arms at 3 months was 3.00 (95% CI 0.06–5.93; p=0.05), indicating significant superiority of SC. Within the SC+COSI arm, children showed a mean 16.5-minute reduction in sleep onset latency and parents increased their knowledge.<br>• Only 23 (53%) families accessed the core materials.<br>• Incremental mean cost of SC+COSI was £1,232 (95% credibility interval £535–£3,455) with a mean incremental Quality Adjusted Life Year (QALY) of 0.00 (95% CI -0.03 to 0.04), yielding an incremental cost-effectiveness ratio of £433,167 per QALY gained a (0.04 probability of being cost-effective at the £30,000/QALY threshold).
**Conclusions**	• Improved objective sleep onset latency and enhanced parental knowledge suggest that the underlying behaviour change techniques hold value.
