# Supplementary material for: ‘I Didn't Even Associate the Two Together at All’: A Qualitative Study of ‘Information Work’ Undertaken by Parents and Their Children With Epilepsy to Make Sense of Sleep and Seizures
Source: Health Expect. 2026 Jul 14;29(4):e70763. doi: 10.1111/hex.70763 (PMC13366387; doi:10.1111/hex.70763)
Supplement: Supplementary file 3 — Supporting File 3 [file HEX-29-e70763-s002.pdf]

Trust/Site Address 1 | Trust/Site Address 1 | Postcode | Telephone Number: 00000 000 000

An overview of the interview topics and questions for families using COSI
(CASTLE Online Sleep Intervention)

Thank you for your interest in taking part in an interview for the CASTLE Sleep-E study.

The CASTLE-SLEEP-E trial aims to find out whether giving families access to an online sleep intervention (known as the CASTLE Online Sleep Intervention or "COSI" for short) will help improve their quality of sleep. The information given in COSI is personalised and has been designed specifically for parents/carers of children with epilepsy.

The questions we are planning to ask have been developed with the support of parents/carers of children with epilepsy. They suggested that it would be helpful for parents/carers to see the sorts of questions the researchers would be asking before the interview, to help them think about the things they might want to tell the researcher

We may not ask all of the questions listed below, nor in the order that they appear, and some areas may be discussed in more depth than others.

The researcher who does the interview will be sensitive about the questions you may find upsetting (for example, questions about what your fears are about your child's epilepsy).

We are really interested in where you get your information from (e.g., Google, your friends, your doctor).

We are also really interested in who makes the decisions about your child's epilepsy (e.g., mostly just one person, a joint effort between the adults in your family, whether your child is usually involved or not involved, or whether it depends on the decision).

We will be asking about the same things at both the first and second interviews, although the focus in the second interview will be on what, if anything has changed, and how and why.

Please tell me about you, your family's and your child's experiences of living with epilepsy

In this question we are likely to ask you about

- Changes to family life, your child's life and school life.
- The management/treatment of your child's seizures.
- Whether your child's seizures make you anxious or frightened (if so, what are you anxious about).
- Whether your feelings about your child's epilepsy have changed over time.
- Where you get your information from.
- Who makes the decisions.

Things I might want to talk about in the interview

Please tell me about any issues you have with your own and your child's sleep

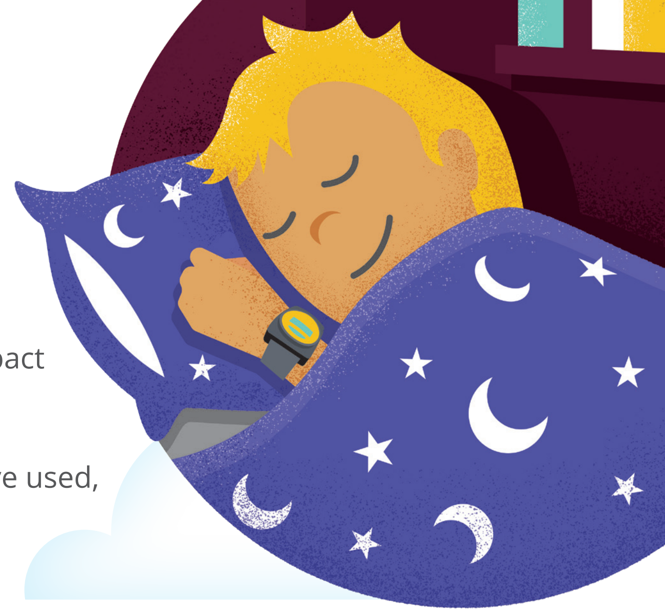

In this question we are likely to ask you about

- What bedtime is like.
- How important you think sleep is.
- Disruptions to your own and your child's sleep and the impact that this may have on your child, yourself and your family.
- What strategies or things you have used (apart from COSI) to help improve sleeping (for example, apps you might have used, your child sleeps with you).
- Where you get your information from.
- Who makes the decisions.

Things I might want to talk about in the interview

Please tell me about you and your child's experience of using COSI

In this question we are likely to ask you about

- How you and your child found using the sleep monitor.
- How motivated and committed you were in using COSI.
- What made it easy or difficult to use/stick to using COSI.
- What bits of COSI you liked the most and what bits you didn't like so much.
- What we could do to make COSI work better.
- Where you get your information from.
- Who makes the decisions.

Things I might want to talk about in the interview

Anything else you want to tell me?

Other things I might want to talk or ask about in the interview

Trust/Site Address 1 | Trust/Site Address 1 | Postcode | Telephone Number: 00000 000 000

An overview of the interview topics and questions for usual care families

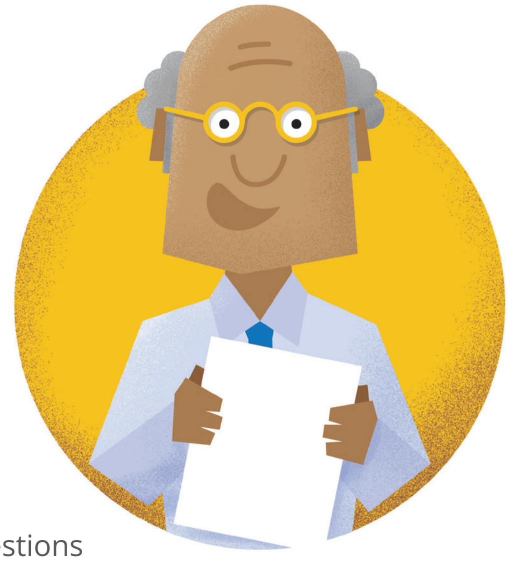

Thank you for your interest in taking part in an interview for the CASTLE Sleep-E study. The questions we are planning to ask have been developed with the support of parents/carers of children with epilepsy. They suggested that it would be helpful for parents/carers to see the sorts of questions the researchers would be asking before the interview, to help them think about the things they might want to tell the researcher.

We may not ask all of the questions listed below, nor in the order that they appear, and some areas may be discussed in more depth than others.

The researcher who does the interview will be sensitive about the questions you may find upsetting (for example, questions about what your fears are about your child's epilepsy).

We are really interested in where you get your information from (e.g., Google, your friends, your doctor).

We are also really interested in who makes the decisions about your child's epilepsy (e.g., mostly just one person, a joint effort between the adults in your family, whether your child is usually involved or not involved, or whether it depends on the decision).

We will be asking about the same things at both the first and second interviews, although the focus in the second interview will be on what, if anything has changed, and how and why.

Please tell me about you, your family's and your child's experiences of living with epilepsy

In this question we are likely to ask you about

- Changes to family life, your child's life and school life.
- The management/treatment of your child's seizures.
- Whether your child's seizures make you anxious or frightened (if so, what are you anxious about).
- Whether your feelings about your child's epilepsy have changed over time.
- Where you get your information from.
- Who makes the decisions.

Things I might want to talk about in the interview

____

____

____

Please tell me about any issues you have with your own and your child's sleep

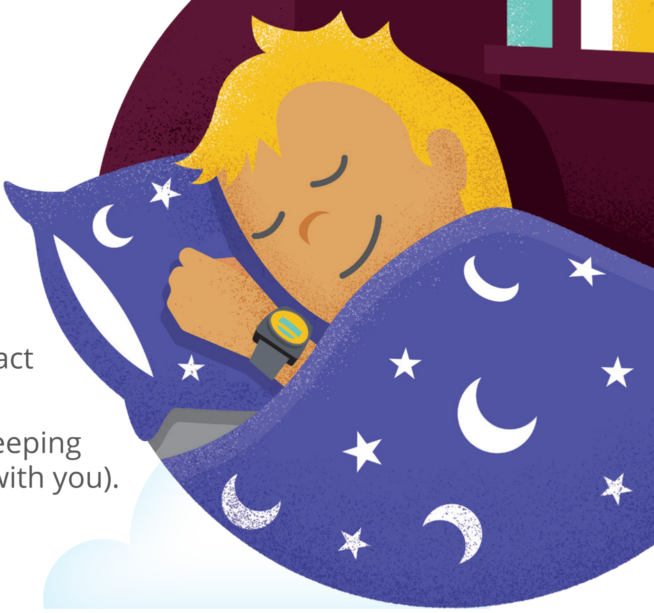

In this question we are likely to ask you about

- What bedtime is like.
- How important you think sleep is.
- Disruptions to your own and your child's sleep and the impact that this may have on your child, yourself and your family.
- What strategies or things you have used to help improve sleeping (for example, apps you might have used, your child sleeps with you).
- Where you get your information from.
- Who makes the decisions.

Things I might want to talk about in the interview

Please tell me about you and your child's experience of using the sleep monitor

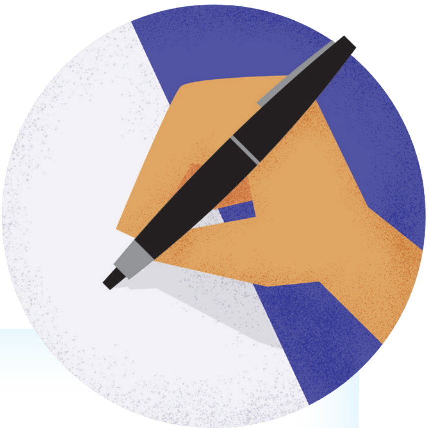

In this question we are likely to ask you about

- How you and your child found using the sleep monitor
- Where you get your information from.
- Who makes the decisions.

Things I might want to talk about in the interview

Anything else you want to tell me?

Other things I might want to talk or ask about in the interview

CASTLE SLEEP-E

Children & Young People's Activity Booklet

The questions in this booklet are the things we want talk about in the interview.

This is me!

Please draw a picture or write something to tell us about who you are and the things you like to do.

How I feel about my epilepsy

Please circle the emoji which shows how you feel about your epilepsy.

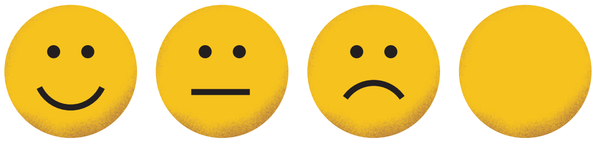

Or draw your own!

One word I would use to say how I feel about my epilepsy is....

....

Does your epilepsy change what you do with your friends?

Circle the words which show what you think.

No, not at all **A little bit** **Yes, quite a lot**

Does having epilepsy change anything for you at school?

Circle the words which show what you think.

No, not at all **A little bit** **Yes, quite a lot**

Can you tell us more?

Think about any changes that having epilepsy makes when you are with your **friends** or at **school**.

....

....

....

How well do you usually sleep?

Circle the words which show how well you sleep.

Not very well **Just okay** **Very well**

Can you tell us more?

Think about going to sleep, staying asleep and getting up in the morning.

....

....

....

How easy do you find it to...?

Circle the stars (1 star is very hard, 5 stars is very easy).

	Very hard				Very easy
**Go to sleep**	★	★★	★★★	★★★★	★★★★★
**Stay asleep**	★	★★	★★★	★★★★	★★★★★
**Wake up in the morning**	★	★★	★★★	★★★★	★★★★★

What things help you to get to sleep?

....

....

Please tell us which is most important?

....

....

Who usually makes the decisions about going to bed?

Circle your answers.

Me **My mum/dad/carer** **We decide together**

How does that make you feel?

Circle your answer or write in the space.

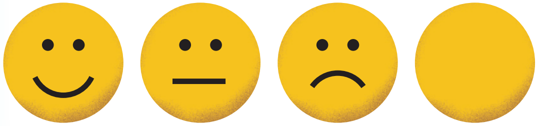

Or draw your own!

....

As part of the CASTLE Sleep-E study you had to wear a sleep watch.

How did you feel about wearing the watch?

Circle the emoji which shows how you feel.

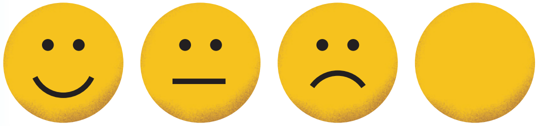

Or draw your own!

One word I would use to say how I felt about wearing the watch is... ....

Your mum/dad/carer has been using COSI to help you sleep better.

What do you think about the things your mum/dad/carer has being doing?

Please circle any words to show what you think about COSI

Helpful **Takes too much time**
Boring **Annoying** **Can't remember**
Enjoyable **Good** **Bad** **Worrying**

Please can you ask your mum/dad/carer to take a picture/scan of this booklet and send it to **castle@edgehill.ac.uk**

CASTLE SLEEP-E

NHS

Children & Young People's

Activity Booklet

The questions in this booklet are the things we want talk about in the interview.

This is me!

Please draw a picture or write something to tell us about who you are and the things you like to do.

How I feel about my epilepsy

Please circle the emoji which shows how you feel about your epilepsy.

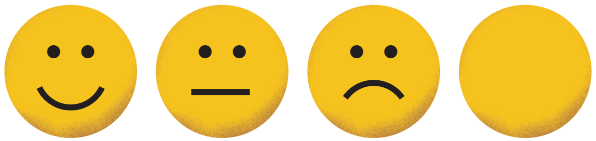

Or draw your own!

One word I would use to say how I feel about my epilepsy is....

....

Does your epilepsy change what you do with your friends?

Circle the words which show what you think.

No, not at all **A little bit** **Yes, quite a lot**

Does having epilepsy change anything for you at school?

Circle the words which show what you think.

No, not at all **A little bit** **Yes, quite a lot**

Can you tell us more?

Think about any changes that having epilepsy makes when you are with your **friends** or at **school**.

....

....

....

How well do you usually sleep?

Circle the words which show how well you sleep.

Not very well **Just okay** **Very well**

Can you tell us more?

Think about going to sleep, staying asleep and getting up in the morning.

....

....

....

How easy do you find it to...?

Circle the stars (1 star is very hard, 5 stars is very easy).

	Very hard				Very easy
**Go to sleep**	★	★★	★★★	★★★★	★★★★★
**Stay asleep**	★	★★	★★★	★★★★	★★★★★
**Wake up in the morning**	★	★★	★★★	★★★★	★★★★★

What things help you to get to sleep?

....

....

Please tell us which is most important?

....

....

Who usually makes the decisions about going to bed?

Circle your answers.

Me **My mum/dad/carer** **We decide together**

How does that make you feel?

Circle your answer or write in the space.

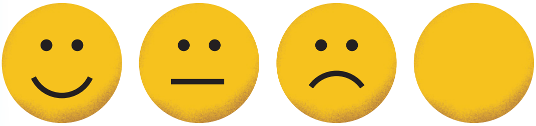

Or draw your own!

....

As part of the CASTLE Sleep-E study you had to wear a sleep watch.

How did you feel about wearing the watch?

Circle the emoji which shows how you feel.

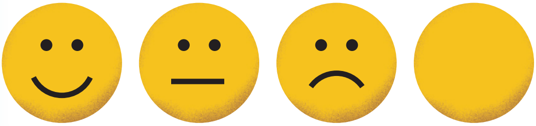

Or draw your own!

One word I would use to say how I felt about wearing the watch is... ....

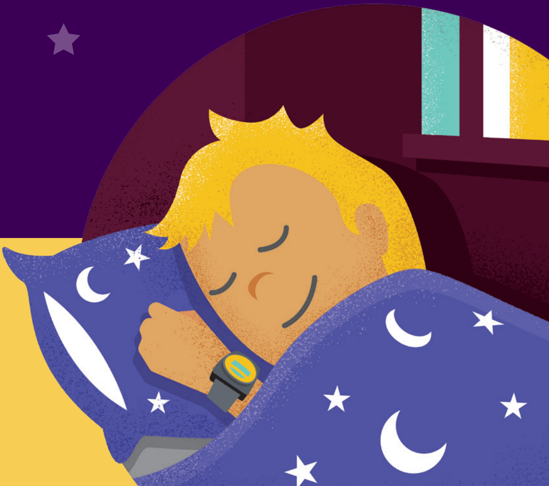

Please can you ask your mum/dad/carer to take a picture/scan of this booklet and send it to **castle@edgehill.ac.uk**

CASTLE Sleep-E C&YP Activity Booklet (Usual Care), V1.0, 24/03/2021 IRAS: 289580
