# Supplementary material for: ‘I Didn't Even Associate the Two Together at All’: A Qualitative Study of ‘Information Work’ Undertaken by Parents and Their Children With Epilepsy to Make Sense of Sleep and Seizures
Source: Health Expect. 2026 Jul 14;29(4):e70763. doi: 10.1111/hex.70763 (PMC13366387; doi:10.1111/hex.70763)
Supplement: Supplementary file 4 — Supporting File 4 [file HEX-29-e70763-s001.pdf]

Interview Guide

Prior to the interviews (as appropriate)

- The researcher will introduce themselves and check the parent's/child's understanding of the study.
- The researcher will remind the parent/child that, ideally, they would take part in two interviews (the first at 3 months and the second at 6 months after they entered the trial).
- The researcher will provide the opportunity for the parent/child to ask questions and/or clarify their involvement. At both interviews, the researcher will gain ongoing verbal confirmation of consent/assent.
- The parent/child will be made aware/reminded that the interview will be audio recorded.
- The parent/child will be made aware/reminded that the interview can be paused, stopped or discontinued at any time and that there are no right or wrong answers to the questions asked.
- Parents/children who have given consent/assent will be reminded that they can discontinue their participation in the interview part of the CASTLE Sleep-E study at any point.

NOTES FOR RESEARCHERS:

- **Questions/prompts must be tailored to each individual participant.**
- **Particular sensitivity must be taken with questions relating to parents' fears, dying, emotions etc**
- **Simpler wording will be used when working with children.**

The Topic Areas and Prompts

The broad topic areas and prompts have been presented but these will not necessarily be asked verbatim or in the order (within each interview) as indicated below.

The same topics will be asked at both the first and second interviews, although the focus in the second interview will be on what, if anything has changed, and how and why.

The parents and children will be encouraged to talk freely and lead the direction of the interview. The children's interviews will be guided partly by the information they give in the activity booklets/sheets.

Please tell me about you, your family's and your child's experiences of living with (Rolandic[1]) epilepsy *(NOTE: Question and all prompts appropriate for COSI and Usual Care parents)*

- Changes to family life, your child's life and school life.
- The management/treatment of your child's seizures.
- Whether your child's seizures make you anxious or frightened (if so, what are you anxious about; this may include fear of child dying).
- Whether your feelings about your child's epilepsy have changed over time.
- Where you get your information from.
- Who makes the decisions (power, positionality, willingness to share decisions)?

[1] Note: initial plans for the trial has been to focus on Rolandic epilepsy but this was changed in response to recruitment challenges within the trial.

Please tell me about any issues you have with your own and your child's sleep *(NOTE: Question and all prompts appropriate for COSI and Usual Care parents, apart from reference to 'apart from COSI' in bullet point 4 which is only for COSI parents)*

- What bedtime is like.
- How important you think sleep is.
- What disruptions to your own and your child's sleep and the impact that this may have on your child, yourself and your family.
- What strategies or things you have used (apart from COSI) to help improve sleeping (for example, apps you might have used, your child sleeps with you).
- Where you get your information from.
- Who makes the decisions (power, positionality, willingness to share decisions)?

Please tell me about you and your child's experience of using COSI *(NOTE: Question and all prompts only appropriate for COSI parents)*

- How you and your child found using the sleep monitor.
- How motivated and committed you were in using COSI.
- What made it easy or difficult to use/stick to using COSI.
- What bits of COSI you liked the most and what bits you didn't like so much.
- What we could do to make COSI work better.
- Where you get your information from.
- Who makes the decisions (power, positionality, willingness to share decisions)?

Please tell me about you and your child's experience of using the sleep monitor *(NOTE: This question is for Usual Care parents)*

- How you and your child found using the sleep monitor
- Where you get your information from.
- Who makes the decisions (power, positionality, willingness to share decisions)?

Anything else you want to tell me?

Closing the interview

- The parent/child will be thanked and made aware that they can ask for a summary of the key points from their own interview within seven days and reminded that they have up to seven days to withdraw their data.
- The parent/child will be offered the opportunity to ask any questions if they have them.
- The child will be given a certificate of thanks after each interview.
- The parent will be given the Helpful Information sheet.
