# Supplementary material for: ‘I Didn't Even Associate the Two Together at All’: A Qualitative Study of ‘Information Work’ Undertaken by Parents and Their Children With Epilepsy to Make Sense of Sleep and Seizures
Source: Health Expect. 2026 Jul 14;29(4):e70763. doi: 10.1111/hex.70763 (PMC13366387; doi:10.1111/hex.70763)
Supplement: Supplementary file 5 — Supporting File 5 [file HEX-29-e70763-s003.pdf]

Supporting file 5: Overview of sleep measure

We calculated a Composite Sleep Disturbance Index (CSDI) to give an indication of the sleeplessness problems in the sample. This measure is based on allocating scores according to the frequency and severity of sleeplessness problems as reported by parents in the Child Sleep Habits Questionnaire (CSHQ) (outcome measure used in the trial). Settling (2 items), night-waking (2 items), early-morning waking (1 item) and co-sleeping (2 items) were assigned scores for frequency as follows: 0-1 times per week = 0, 2-4 times per week = 1 and 5-7 times per week = 2. Item scores were summed with scores ranging from 0-14, with higher scores indicating increased problems.

In line with Montgomery, Wiggs and Stores (2004), a score of 7 or more would be considered to indicate the presence of at least mild sleeplessness. For this sample the mean score (and standard deviation (sd)) was 5.9 (sd 3.6), with scores ranging from 2-14 and 6 children scored at or above the threshold for mild sleeplessness (indicating that 16 children did not have significant sleeplessness problems).

Reference

Montgomery P, Stores G and Wiggs L (2004) The relative efficacy of two brief treatments for sleep problems in young learning disabled (mentally retarded) children: a randomised controlled trial. *Archives of Disease in Childhood* 89(2): 125–130.
